# The Protective Effects of Sivelestat Sodium on the Basis of Corticosteroid Therapy in Patients With Moderate-to-Severe Acute Respiratory Distress Syndrome

**DOI:** 10.1155/emmi/1824299

**Published:** 2025-02-12

**Authors:** Yujie Ma, Guofu Tang, Xiaotong Liu, Qiang Gao

**Affiliations:** ^1^Department of Cardiovascular Medicine, Dazhou Dachuan District People's Hospital (Dazhou Third People's Hospital), Dazhou, China; ^2^Department of Critical Care Medicine, Dazhou Central Hospital, Dazhou, China; ^3^Department of Clinical Medicine, North Sichuan Medical College, Nanchong, China

**Keywords:** ARDS, corticosteroids, mortality, risk factors, sivelestat

## Abstract

**Objective:** We aimed to evaluate the protective effects of sivelestat sodium on the basis of corticosteroid therapy in patients with moderate-to-severe acute respiratory distress syndrome (ARDS).

**Methods:** We retrospectively investigated 127 patients with confirmed moderate-to-severe ARDS treated in the intensive care unit (ICU) at Dazhou Central Hospital. Patients were divided into the control group (corticosteroids alone) and the combination therapy of steroids and sivelestat sodium (CTSSS) group according to the therapeutic interventions. The primary outcome was in-hospital mortality. And the baseline characteristics and laboratory findings of patients were collected for analysis.

**Results:** The overall mortality rate in 127 patients was 48.8%. There was no statistically significant difference in in-hospital mortality between the CTSSS group and the control group (45.3% vs. 56.1%). In the subgroup of patients aged < 80 years or with an Acute Physiology and Chronic Health Evaluation (APACHE) II score < 30, CTSSS could reduce the risk of mortality (odds ratio [OR] = 0.41, 95% confidence interval [CI], 0.17–0.96, *p*=0.041; OR = 0.31, 95% CI, 0.13–0.77, *p*=0.012; respectively). Among patients aged 80 years or older, those with CTSSS exhibited a significantly elevated risk of mortality (OR = 13; 95% CI, 1.20–140.73; *p*=0.035).

**Conclusion:** Compared with corticosteroids alone, CTSSS could improve oxygenation index, increase lymphocyte count, protect extrapulmonary organs and reduce in-hospital mortality rate in patients with moderate-to-severe ARDS in specific subgroups (age < 80 years or APACHE II score < 30). It might be advisable to avoid CTSSS in moderate-to-severe ARDS patients aged 80 years or older. Prospective studies involving larger sample sizes are needed to verify these findings.

## 1. Introduction

Acute respiratory distress syndrome (ARDS) is a common and potentially fatal clinical syndrome in the intensive care unit (ICU), which is characterised by acute hypoxaemic respiratory failure with bilateral infiltrates on chest imaging in the absence of cardiac failure and fluid overload [1, 2]. Despite advances in lung protective ventilation strategies [3–6], extracorporeal support [7–9] and other comprehensive management, the mortality in patients with ARDS remains to be as high as 29%–45% [10–12].

As potent anti-inflammatory drugs, the use of corticosteroids in ARDS has always been controversial. However, more and more randomised control trials (RCTs) suggest that the use of corticosteroids probably improves outcomes of ARDS [13]. And some guidelines have started advocating for the use of corticosteroids in ARDS [14], especially in patients with current coronavirus disease 2019 (COVID-19) ARDS [15]. Thus, early corticosteroid therapy has been recommended for moderate-to-severe ADRS in clinical practice [16, 17].

Sivelestat sodium, as an inhibitor of neutrophil elastase (NE), has already been approved for clinical use to treat ARDS in China since March 2020. Similar to the role of corticosteroids in ARDS management, sivelestat demonstrates obvious anti-inflammatory and lung protective effects in animal models of treating acute lung injury [18–21], as well as in clinical studies [22, 23]. Currently, although the clinical efficacy of sivelestat remains controversial [24], sivelestat sodium has been used in patients with ARDS and also for ARDS requiring corticosteroid therapy in China. Notably, the combination therapy of steroids and sivelestat sodium (CTSSS) in patients with moderate-to-severe ARDS has not been reported before. The safety of using the two anti-inflammatory drugs with different mechanisms simultaneously for moderate-to-severe ARDS is still unclear. And it is unknown whether sivelestat sodium retains protective effects on the basis of corticosteroid therapy in adults with moderate-to-severe ARDS in clinical practice.

Therefore, in this study, we aimed to investigate the protective effects of sivelestat sodium on the basis of corticosteroid therapy in patients with moderate-to-severe ARDS by longitudinal comparison. In addition, we tried to identify the potential beneficiary or victims due to sivelestat sodium in the study population in order to better guide treatment decision-making.

## 2. Materials and Methods

### 2.1. Study Design

We conducted this longitudinal and retrospective cohort study at Dazhou Central Hospital, a Chinese teaching hospital in Sichuan province, between May 2021 and May 2023. We started to use sivelestat sodium for ARDS in our ICU after February 2022. Adult patients (≥ 18 years) who were admitted to the comprehensive ICU with confirmed moderate-to-severe ARDS (PaO2/FiO2 < 200 mmHg) were consecutively included. The diagnosis of ARDS followed the Berlin definition [1]. The patients were excluded based on the following criteria: (1) patients who did not use corticosteroids because of suspected or confirmed gastrointestinal bleeding; (2) patients who died or were discharged within 48 h after ICU admission; (3) patients whose medication time of steroids or sivelestat sodium was less than 2 days; and (4) patients whose data were incomplete.

Eventually, patients enrolled in this study were divided into two groups according to the date, February 2022, when we started to use sivelestat sodium. Before February 2022, patients treated with corticosteroids alone were enrolled in the control group. And after February 2022, patients receiving CTSSS were included in the CTSSS group. Sivelestat sodium, which was diluted in normal saline to a total volume of 48 mL, was pumped by a 24-h continuous intravenous administration at a dose of 0.2 mg/kg/hour. The dosage of methylprednisolone used in this study was 1 mg/kg/day. The longest duration of sivelestat sodium and methylprednisolone was 14 days. The timing of initiating methylprednisolone or sivelestat sodium administration was within 24 h of diagnosing moderate-to-severe ARDS. In addition, all patients in both groups received invasive mechanical ventilation but no patient received extracorporeal membrane oxygenation.

We reviewed all medical records and collected the following data: demographics, comorbidities, the aetiology of ARDS, the latest laboratory findings when initiating corticosteroid therapy or CTSSS, the latest laboratory findings when discontinuing corticosteroid therapy or CTSSS, Acute Physiology and Chronic Health Evaluation (APACHE) II scores, Sequential Organ Failure Assessment (SOFA) scores, complications and clinical outcomes. This study was approved by the Ethics Committee of Dazhou Central Hospital (No. 2024042). The informed consent was waived because of the retrospective and non-interventional nature of the study.

### 2.2. Definitions

Septic shock was defined if patients required a vasopressor to uphold the mean arterial pressure (MAP) ≥ 65 mmHg and serum lactate > 2 mmol/L despite sufficient fluid resuscitation [25].

ΔLaboratory finding (such as Δwhite blood cell count) was defined as the difference between the latest laboratory findings when discontinuing corticosteroid therapy or CTSSS and the latest laboratory findings when initiating corticosteroid therapy or CTSSS.

### 2.3. Statistical Analysis

We used software package SPSS 22.0 for Windows (SPSS Inc., Chicago, IL, United States) to perform all statistical procedures. Continuous variables were tested for normality using the Shapiro–Wilk test. Data with normal distribution were expressed as mean ± standard deviation (SD) and compared using Student's *t*-test. Data with skewed distribution were described using median and interquartile range (IQR) values and were compared by the Mann–Whitney *U* test. Categorical variables were compared by chi-square test or Fisher's exact test. Logistic regression was used for subgroup analysis by the R version 4.2.3 software and risk factor analysis by SPSS software. *p* < 0.05 was considered statistically significant.

## 3. Results

### 3.1. Patients

**Figure 1:**
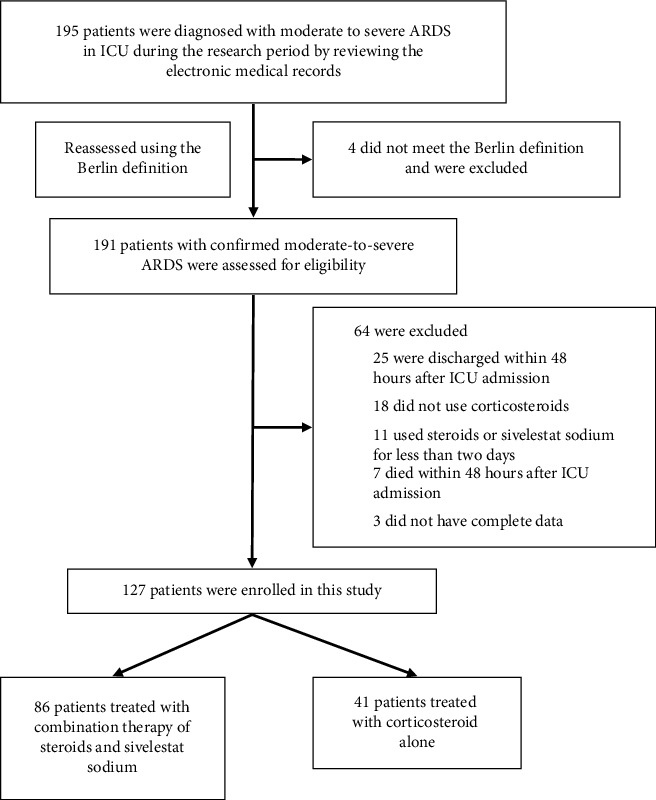
Flow chart of patient enrolment.

During the 2-year study period, a total of 191 patients were diagnosed with moderate-to-severe ARDS.  Figure 1 illustrates the detailed process of enrolment and exclusion. Ultimately, 127 patients with confirmed moderate-to-severe ARDS were enrolled. Eighty-six patients were treated with CTSSS, and 41 patients were treated with corticosteroids alone.

The baseline characteristics of 127 patients are shown in Table 1. The median age was 71 years (IQR, 59–78; range, 24–91 years), and only 28 patients (22.0%) were women. The main reasons of ARDS were pneumonia (85 [66.9%]) and sepsis (27 [21.3%]). The proportion of COVID-19 was 42.5% (54/127). Of 127 moderate-to-severe ARDS, the overall in-hospital mortality rate was 48.8% (62/127).

### 3.2. Comparisons Between the CTSSS Group and the Control Group

Table 1 shows that the differences in baseline characteristics between the CTSSS group and the control group were not statistically significant. And there was no statistically significant difference in baseline laboratory findings when initiating corticosteroid therapy or CTSSS between the two groups (Table 2). Thus, the comparisons presented in Tables 1 and 2 indicated that there was no statistical heterogeneity between the two groups at baseline (all *p* > 0.05).

Compared with the control group, the median of Δpartial pressure of oxygen/fraction of inspired oxygen (PaO2/FiO2) (100 vs. 64, *p*=0.075) and Δlymphocyte count (0.08 vs. −0.06, *p*=0.095) in the CTSSS group when discontinuing corticosteroid therapy or CTSSS were higher (Table 3), but the differences were not statistically significant. Furthermore, there were no statistically significant differences in in-hospital mortality, length of invasive mechanical ventilation, length of ICU stay, length of hospital stay and duration of shock between the CTSSS group and the control group (Table 4). Compared with the corticosteroids alone, CTSSS did not improve the outcomes of patients with moderate-to-severe ARDS.

### 3.3. Subgroup Analysis

**Figure 2:**
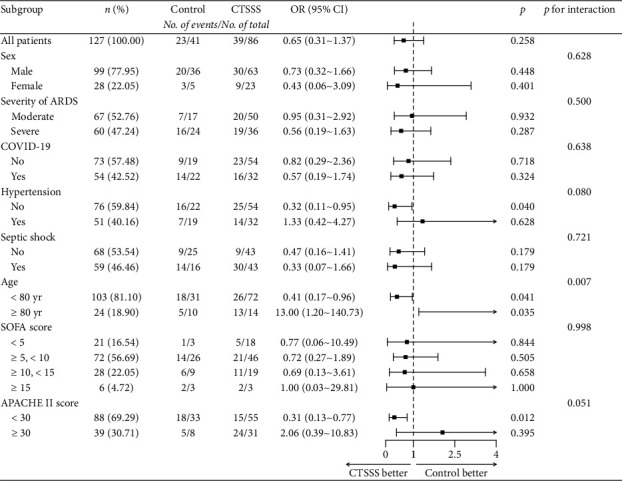
Effects of CTSSS on in-hospital mortality in specific subgroups. CTSSS, combination therapy of steroids and sivelestat sodium; OR, odds ratio; ARDS, acute respiratory distress syndrome; COVID-19, current coronavirus disease 2019; APACHE, Acute Physiology and Chronic Health Evaluation; SOFA, Sequential Organ Failure Assessment.

Exploratory analysis (Figure 2) revealed that the effects of CTSSS strategy on mortality interacted with age and APACHE II score (*p*=0.007 for the interaction and *p*=0.051 for the interaction, respectively). In the CTSSS group, the death risk of patients under the age of 80 years decreased by 59% compared with that in the control group (odds ratio [OR] = 0.41; 95% confidence interval [CI], 0.17–0.96; *p*=0.041). On the contrary, patients aged 80 years or older in the CTSSS group demonstrated a higher death risk (OR = 13; 95% CI, 1.20–140.73; *p*=0.035). Additionally, patients with an APACHE II score below 30 in the CTSSS group demonstrated a significantly decreased death risk (OR = 0.31; 95% CI, 0.13–0.77; *p*=0.012). The major differences in changes of laboratory findings are shown in Table 5, and more details are shown in Table S1 (in the Supporting Appendix). Higher medians of ΔPaO2/FiO2 and Δlymphocyte count were observed in patients under 80 years old in the CTSSS group (*p*=0.016, *p*=0.016, respectively; Table 5). Similar trends were observed in patients with an APACHE II score below 30 in the CTSSS group, but the difference was not statistically significant. However, a significantly lower median of ΔNT-proBNP was observed in the same population (*p*=0.006; Table S1). Moreover, a lower median of Δlymphocyte count was observed in patients aged 80 years or older who received CTSSS, but the difference was also not statistically significant. As shown in Table 4, patients under 80 years old in the CTSSS group had a significantly lower incidence rate of gastrointestinal bleeding and in-hospital mortality rate (*p*=0.025, *p*=0.039, respectively). And patients with an APACHE II score below 30 who received CTSSS had lower in-hospital mortality rate (*p*=0.011).

### 3.4. Assessment of Risk Factors for Overall Mortality

Univariate analysis (Table S2 in the Supporting Appendix) showed that overall mortality in this study was significantly associated with higher age (*p*=0.005), severe ARDS (*p*=0.042), higher APACHE II score (*p* < 0.001), higher SOFA score (*p*=0.009), chronic kidney disease (*p*=0.008), septic shock (*p* < 0.001), decreased ΔPaO2/FiO2 (*p* < 0.001), decreased Δlymphocyte count (*p*=0.001), decreased Δplatelet count (*p* < 0.001), increased Δdirect bilirubin (*p*=0.019), increased Δaspartate aminotransferase (*p*=0.008), increased Δcreatinine (*p*=0.001), increased ΔN-terminal prohormone of brain natriuretic peptide (NT-proBNP) (*p*=0.001), increased ΔC-reactive protein (*p*=0.008), increased Δprocalcitonin (*p* < 0.001) and increased ΔSOFA score (*p* < 0.001). The prolonged duration of sivelestat sodium (*p*=0.038) might be a protective factor for overall mortality. And a higher median of duration of corticosteroids was observed in the survival group, but the difference between the survival group and the death group was not statistically significant.

All variables in Table S2 were included in the logistic regression analysis in order to avoid missing important risk factors. Based on univariate analysis, further logistic regression analysis showed that prolonged duration of corticosteroids (*p*=0.014), increased ΔSOFA score (*p* < 0.001), higher APACHE II score (*p*=0.001) and septic shock (*p*=0.004) were independent risk factors for overall mortality (Table 6). And the prolonged duration of sivelestat sodium (*p*=0.004) remained to be a protective factor for overall mortality in this study.

## 4. Discussion

ARDS commonly contributes to the mortality of critically ill patients of all ages. While patients with ARDS in third-world countries have experienced an unacceptably high hospital mortality rate of 50% [26], the United States continues to struggle with an in-hospital mortality as high as 39% [27]. Compared with non-ARDS patients, the mortality rate of ARDS patients increases by 15% in ICU [28]. In this study, the in-hospital mortality of moderate-to-severe ARDS in ICU was 48.8%. The mortality rates of moderate ARDS and severe ARDS were 43.5% and 56.5%, respectively. The LUNG SAFE study reported a hospital mortality rate of 40.3% for patients with moderate ARDS [10]. And the mortality rate of 46.1% in patients with severe ARDS is lower than the mortality rate of 56.5% in our study. It should be noted that our study was conducted during the COVID-19 pandemic and the proportion of ARDS due to COVID-19 was 42.5% (54/127). As a result, it is possible that the incidence and outcomes of ARDS change over time [2].

The purpose of the present study was to investigate whether sivelestat sodium had protective effects on the basis of corticosteroid therapy in patients with moderate-to-severe ARDS. Our findings suggested that sivelestat sodium could not further improve the overall prognosis of moderate-to-severe ARDS. But compared with the corticosteroid therapy alone, CTSSS could reduce the mortality rate in specific subgroups (age < 80 yr or APACHE II score < 30). Interestingly, CTSSS could not only improve oxygenation but also might increase the lymphocyte count in the above two subgroups. In addition, CTSSS might reduce the incidence of gastrointestinal bleeding and mitigate cardiac dysfunction in the specific subgroups. ARDS is heterogeneous, so the pathophysiological conditions and responses to treatment may be heterogeneous, and the effectiveness of sivelestat may be shown in specific clinical conditions [29]. In this study, logistic regression analysis showed that prolonged duration of corticosteroids, increased ΔSOFA score, higher APACHE II score and septic shock were independent risk factors for overall mortality. These factors might influence the efficacy of sivelestat. A recent study has revealed that the efficacy of sivelestat was correlated with age, cancer, haemodialysis and use of methylprednisolone [30]. Further, logistic regression analysis in this study also proved that the prolonged duration of sivelestat sodium was a protective factor for survival in patients with moderate-to-severe ARDS. A retrospective data analysis found that sivelestat was an independent predictor of survival with sepsis complicated by ARDS and disseminated intravascular coagulation [31].

On the other hand, patients aged 80 years or older and were treated with CTSSS showed higher death risk in the present study. And a lower median of Δlymphocyte count was observed in this subgroup although the difference was not statistically significant. Neutrophils, which can release neutrophil extracellular traps, elastase and result in increased permeability of the membrane, play an important role in the acute inflammation of ARDS [29, 32]. NE can promote the development of ARDS by activating and processing proinflammatory cytokines [33, 34]. Sivelestat is a selective NE inhibitor, which can inhibit the neutrophil activation and reduce the inflammation in the lungs [18–23]. Perhaps, this anti-inflammatory effect may also protect extrapulmonary organs such as gastrointestinal tract and heart. A recent study showed that sivelestat sodium might improve gastrointestinal dysfunction, alleviate deregulated inflammation and decrease the severity of illness in septic patients [35]. Sivelestat sodium also showed myocardial protective effect in rat models with sepsis-induced myocarditis [36]. Meanwhile, NE is essential for the defence of host [37]. Excessive anti-inflammatory measures may lead to a decline in host immunity, especially for patients aged 80 years or older whose immunity may be already not good. Hence, the trend of Δlymphocyte count in patients aged 80 years or older introduced the doubt whether CTSSS might inhibit the host immune defence associated with lymphocyte. Of course, more studies are needed to clarify these potential mechanisms.

Although the difference between the survival group and the death group was not statistically significant, a higher median (6 days) of the duration of corticosteroids was observed in the survival group in this study. A meta-analysis suggested that patients with ARDS who received a longer duration of corticosteroids (over 7 days) had higher rates of survival [13]. Recently, a multicentre, randomised controlled trial showed that early administration of high-dose (20 mg once daily) dexamethasone for 5 days followed by low-dose (10 mg once daily) dexamethasone for 5 days could shorten the duration of mechanical ventilation and reduce overall mortality in patients with moderate-to-severe ARDS [17]. As mentioned previously, our findings displayed that the prolonged duration of corticosteroids was an independent risk factor for overall mortality in patients with moderate-to-severe ARDS. Therefore, we believe that prolonged duration of corticosteroids may be more beneficial for ARDS, but there will be an upper limit on the duration of administration.

The strengths of this study were as follows: (1) the research only focused on moderate-to-severe ARDS in our comprehensive ICU with a relatively large sample, and the management of patients was relatively consistent; (2) sivelestat sodium was almost used in a consecutive series of patients with ARDS when it was recognised by the clinician in our ICU. Therefore, this study was at a low risk of selection bias as far as the use of sivelestat sodium in ARDS patients because that sivelestat sodium had become a routine therapeutic intervention in ARDS patients without being influenced by the subjective wishes of a clinician. And non-significant heterogeneity between the CTSSS group and the control group further confirmed the reliability of the results; (3) the effects of sivelestat sodium on the basis of corticosteroid therapy in ARDS had not been previously reported; (4) subgroup analysis identified the special population that could benefit from sivelestat sodium in patients with moderate-to-severe ARDS.

Of course, this study has several limitations. Firstly, we did not analyse the impact of different types and doses of corticosteroids on mortality in patients with moderate-to-severe ARDS due to the limitation of sample size. Secondly, we did not determine the level of inflammatory cytokine (tumour necrosis factor alpha, interleukin-1β, interleukin-6 and so on) in the management of patients. So it remains unclear whether sivelestat sodium can further reduce cytokine levels on the basis of corticosteroid therapy in adults with moderate-to-severe ARDS. Thirdly, the sequelae of ARDS were not further evaluated because of the absence of a long-term follow-up after discharge. Lastly, 64 patients with moderate-to-severe ARDS were excluded for various reasons in our study, which may have an impact on the assessment of the overall mortality rate.

## 5. Conclusions

The present study suggested that CTSSS could not further reduce the overall mortality rate, length of mechanical ventilation, ICU stay days and duration of shock compared with corticosteroids alone in patients with moderate-to-severe ARDS. However, in the subgroup of age < 80 years or APACHE II score < 30, CTSSS could reduce the risk of death and might improve oxygenation index and increase lymphocyte count. CTSSS may protect extrapulmonary organs such as gastrointestinal tract and heart in specific subgroups. Notably, in the subgroup of age ≥ 80 years, patients with CTSSS demonstrated higher death risk. In addition, the prolonged duration of corticosteroids, increased ΔSOFA score, higher APACHE II score and septic shock were identified as independent risk factors for overall mortality in patients with moderate-to-severe ARDS. Finally, further prospective studies with larger sample sizes are needed to verify these findings.

## Figures and Tables

**Table 1:** Comparison of baseline characteristics between the CTSSS group and the control group.

Parameters	Total (*n* = 127)	CTSSS group (*n* = 86)	Control group (*n* = 41)	*p*
Age, median (IQR) (y)	71 (59–78)	72 (59–77)	67 (58–80)	0.790
Sex				0.064
Male	99 (78.0%)	63 (73.3%)	36 (87.8%)	
Female	28 (22.0%)	23 (26.7%)	5 (12.2%)	
Causes of ARDS				0.682
Pneumonia	85 (66.9%)	54 (62.8%)	31 (75.6%)	
Sepsis	27 (21.3%)	20 (23.3%)	7 (17.1%)	
Surgical operation	6 (4.7%)	5 (5.8%)	1 (2.4%)	
Trauma	5 (3.9%)	4 (4.7%)	1 (2.4%)	
Other causes	4 (3.1%)	3 (3.5%)	1 (2.4%)	
COVID-19	54 (42.5%)	32 (37.2%)	22 (53.7%)	0.080
Severity of ARDS				0.078
Moderate	67 (52.8%)	50 (58.1%)	17 (41.5%)	
Severe	60 (47.2%)	36 (41.9%)	24 (58.5%)	
APACHE II score (mean ± SD)	26.42 ± 8.03	27.28 ± 8.52	24.61 ± 6.64	0.057
SOFA score, median (IQR)	7 (5–10)	7 (5–10)	7 (6–11)	0.150
Duration of corticosteroid, median (IQR) (d)	5 (4–7)	5 (4–7)	6 (4–8)	0.247
Duration of sivelestat sodium, median (IQR) (d)	—	6 (4–8)	—	—
Comorbidities				
Hypertension	51 (40.2%)	32 (37.2%)	19 (46.3%)	0.326
Cardiovascular disease	25 (19.7%)	16 (18.6%)	9 (22.0%)	0.657
Diabetes	24 (18.9%)	17 (19.8%)	7 (17.1%)	0.717
COPD	15 (11.8%)	9 (10.5%)	6 (14.6%)	0.560
Malignancy	12 (9.4%)	8 (9.3%)	4 (9.8%)	> 0.999
Chronic kidney disease	10 (7.9%)	4 (4.7%)	6 (14.6%)	0.075
Bronchiectasia	8 (6.3%)	5 (5.8%)	3 (7.3%)	0.712
Pulmonary aspergillosis	7 (5.5%)	4 (4.7%)	3 (7.3%)	0.680

Abbreviations: APACHE II, Acute Physiology and Chronic Health Evaluation II; ARDS, acute respiratory distress syndrome; COPD, chronic obstructive pulmonary disease; COVID-19, current coronavirus disease 2019; CTSSS, combination therapy of steroids and sivelestat sodium; IQR, interquartile range; SD, standard deviation; SOFA, sequential organ failure assessment.

**Table 2:** Comparison of laboratory findings when initiating corticosteroid therapy or CTSSS.

Parameters, median (IQR)	Total (*n* = 127)	CTSSS group (*n* = 86)	Control group (*n* = 41)	*p*
PaO2/FiO2 (mmHg)	108 (74–152)	111 (70–153)	98 (77–139)	0.444
White blood cell count (× 10^9^/L)	11.99 (7.42–16.52)	11.41 (7.01–17.34)	13.91 (8.86–15.92)	0.399
Neutrophil count (× 10^9^/L)	10.76 (6.34–15.16)	10.41 (6.11–15.84)	12.43 (7.75–14.17)	0.364
Lymphocyte count (× 10^9^/L)	0.46 (0.27–0.70)	0.46 (0.28–0.70)	0.42 (0.27–0.84)	0.687
Haemoglobin, (mean ± SD), (g/L)	111.02 ± 24.51	111.83 ± 26.03	109.32 ± 21.18	0.592
Platelet count (× 10^9^/L)	143 (88–201)	136 (86–192)	147 (90–232)	0.384
Total bilirubin (umol/L)	13.20 (8.69–21.59)	13.80 (9.28–23.13)	11.10 (8.17–17.25)	0.217
Direct bilirubin (umol/L)	5.85 (3.68–10.2)	6.07 (3.40–11.33)	5.70 (3.75–8.90)	0.524
Alanine aminotransferase (U/L)	33 (21–53)	35 (22–57)	33 (18–49)	0.296
Aspartate aminotransferase (U/L)	50 (30–82)	52 (33–85)	49 (29–73)	0.325
Creatinine (μmol/L)	88 (61–150)	87 (60–147)	89 (67–201)	0.511
Albumin (g/L)	30 (26–33)	29 (26–33)	31 (26–33)	0.432
Lactate (mmol/L)	3.26 (2.36–4.36)	3.23 (2.31–4.56)	3.32 (2.43–4.29)	0.693
NT-proBNP (ng/L)	1790 (829–4838)	1870 (629–4388)	1365 (956–6107)	0.914
C-reactive protein (mg/L)	125 (87–184)	126 (86–180)	121 (90–186)	0.703
Procalcitonin (ug/L)	2.54 (0.51–7.86)	2.67 (0.42–8.66)	2.43 (0.53–7.72)	0.965

Abbreviations: CTSSS, combination therapy of steroids and sivelestat sodium; FiO2, fraction of inspired oxygen; IQR, interquartile range; NT-proBNP, N-terminal prohormone of brain natriuretic peptide; PaO2, partial pressure of oxygen; SD, standard deviation.

**Table 3:** Changes in laboratory findings and SOFA score when discontinuing corticosteroid therapy or CTSSS.

Parameters, median (IQR)	Total (*n* = 127)	CTSSS group (*n* = 86)	Control group (*n* = 41)	*p*
ΔPaO2/FiO2 (mmHg)	79 (27–157)	100 (50–167)	64 (19–134)	0.075
ΔWhite blood cell count (× 10^9^/L)	−1.45 (−5.00 to 3.01)	−0.94 (−4.52 to 3.20)	−2.75 (−5.44 to 2.99)	0.386
ΔNeutrophil count, (mean ± SD) (× 10^9^/L)	−0.77 ± 6.65	−0.61 ± 6.40	−1.13 ± 7.20	0.680
ΔLymphocyte count (× 10^9^/L)	0.05 (−0.19 to 0.24)	0.08 (−0.16 to 0.27)	−0.06 (−0.31 to 0.15)	0.095
ΔHaemoglobin, (mean ± SD) (g/L)	−13 ± 20	−12 ± 21	−13 ± 18	0.664
ΔPlatelet count (× 10^9^/L)	−26 (−73 to 17)	−25 (−74 to 16)	−29 (−76 to 35)	0.732
ΔTotal bilirubin (umol/L)	−0.21 (−4.66 to 5.6)	−0.25 (−4.37 to 5.85)	0.40 (−4.89 to 5.31)	0.701
ΔDirect bilirubin (umol/L)	0.30 (−2.02 to 3.01)	0.43 (−1.73 to 3.69)	−0.15 (−2.76 to 2.74)	0.266
ΔAlanine aminotransferase (U/L)	1 (−20 to 12)	2 (−21 to 20)	1 (−18 to 12)	0.891
ΔAspartate aminotransferase (U/L)	−2 (−27 to 25)	−4 (−29 to 38)	−1 (−27 to 15)	0.979
ΔCreatinine (μmol/L)	9 (−11 to 71)	10 (−13 to 72)	9 (−10 to 71)	0.718
ΔAlbumin (g/L)	2.48 (−2.60 to 6.51)	2.63 (−2.55 to 7.37)	1.22 (−2.69 to 6.41)	0.539
ΔLactate (mmol/L)	−0.68 (−1.72 to 0.31)	−0.68 (−1.72 to 0.33)	−0.80 (−1.80 to 0.25)	0.613
ΔNT-proBNP (ng/L)	80 (−1248 to 1618)	0 (−1492 to 1515)	321 (−660 to 2416)	0.265
ΔC-reactive protein (mg/L)	−42 (−98 to −12)	−40 (−91 to −9)	−58 (−117 to −20)	0.283
ΔProcalcitonin (ug/L)	−0.29 (−5.07 to 1.10)	−0.17 (−5.45 to 1.10)	−0.33 (−4.36 to 0.98)	0.867
ΔSOFA score	−1 (−3 to 2)	0 (−3 to 2)	−1 (−3 to 2)	0.679

Abbreviations: CTSSS, combination therapy of steroids and sivelestat sodium; FiO2, fraction of inspired oxygen; IQR, interquartile range; NT-proBNP, N-terminal prohormone of brain natriuretic peptide; PaO2, partial pressure of oxygen; SD, standard deviation; SOFA, sequential organ failure assessment.

**Table 4:** Complications and outcomes in this study.

Stratification	Total	CTSSS group	Control group	*p*
Overall cohort	*n* = 127	*n* = 86	*n* = 41	
Septic shock	59 (46.5%)	43 (50.0%)	16 (39.0%)	0.246
Gastrointestinal bleeding	21 (16.5%)	12 (14.0%)	9 (22.0%)	0.257
Acute renal failure	16 (12.6%)	13 (15.1%)	3 (7.3%)	0.216
Length of IMV, median (IQR) (h)	166 (109–244)	167 (106–245)	166 (112–252)	0.569
Length of ICU stay, median (IQR) (d)	10 (6–13)	10 (6–14)	10 (7–12)	0.887
Length of hospital stay, median (IQR) (d)	13 (8–21)	13 (8–22)	11 (7–19)	0.424
Duration of shock, median (IQR) (d)	2 (0–5)	2 (0–5)	3 (0–6)	0.482
In-hospital death	62 (48.8%)	39 (45.3%)	23 (56.1%)	0.257
Age < 80 yr	*n* = 103	*n* = 72	*n* = 31	
Septic shock	45 (43.7%)	32 (44.4%)	13 (41.9%)	0.814
Gastrointestinal bleeding	17 (16.5%)	8 (11.1%)	9 (29.0%)	0.025
Acute renal failure	10 (9.7%)	8 (11.1%)	2 (6.5%)	0.719
Length of IMV, median (IQR) (h)	180 (117–266)	175 (112–258)	205 (132–305)	0.259
Length of ICU stay, median (IQR) (d)	10 (7–14)	10 (6–14)	10 (7–15)	0.902
Length of hospital stay, median (IQR) (d)	14 (9–21)	15 (10–24)	11 (8–17)	0.121
Duration of shock, median (IQR) (d)	2 (0–5)	2 (0–5)	4 (0–6)	0.184
In-hospital death	44 (42.7%)	26 (36.1%)	18 (58.1%)	0.039
APACHE II score < 30	*n* = 88			
Septic shock	31 (35.2%)	19 (34.5%)	12 (36.4%)	0.863
Gastrointestinal bleeding	12 (13.6%)	6 (10.9%)	6 (18.2%)	0.354
Acute renal failure	7 (8.0%)	6 (10.9%)	1 (3.0%)	0.248
Length of IMV, median (IQR) (h)	182 (132–263)	178 (111–250)	205 (137–307)	0.136
Length of ICU stay, median (IQR) (d)	10 (7–14)	10 (7–14)	10 (7–15)	0.921
Length of hospital stay, median (IQR) (d)	14 (10–22)	14 (10–24)	13 (10–20)	0.283
Duration of shock, median (IQR) (d)	1 (0–4)	1 (0–3)	2 (0–5)	0.056
In-hospital death	33 (37.5%)	15 (27.3%)	18 (54.5%)	0.011

Abbreviations: CTSSS, combination therapy of steroids and sivelestat sodium; ICU, intensive care unit; IMV, invasive mechanical ventilation; IQR, interquartile range.

**Table 5:** Potential differences between the CTSSS group and the control group in specific conditions.

Subgroups	Total	CTSSS group	Control group	*p*
Age < 80 yr	*n* = 103	*n* = 72	*n* = 31	
ΔPaO2/FiO2, median (IQR) (mmHg)	80 (46–164)	107 (55–176)	64 (19–113)	0.016
ΔLymphocyte count, median (IQR) (× 10^9^/L)	0.06 (−0.17 to 0.28)	0.12 (−0.09 to 0.37)	−0.06 (−0.41 to 0.14)	0.016
Age ≥ 80 yr	*n* = 24	*n* = 14	*n* = 10	
Death	18 (75%)	13 (92.9%)	5 (50.0%)	0.050
ΔPaO2/FiO2, median (IQR) (mmHg)	61 (−5 to 142)	60 (−26 to 137)	86 (6–200)	0.429
ΔLymphocyte count, median (IQR) (× 10^9^/L)	−0.05 (−0.26 to 0.13)	−0.07 (−0.39 to 0.01)	0.12 (−0.18 to 0.29)	0.089
APACHE II score < 30	*n* = 88	*n* = 55	*n* = 33	
ΔPaO2/FiO2, median (IQR) (mmHg)	79 (30–163)	107 (59–170)	60 (18–134)	0.089
ΔLymphocyte count, median (IQR) (× 10^9^/L)	0.10 (−0.16 to 0.27)	0.12 (−0.05 to 0.32)	−0.03 (−0.21 to 0.21)	0.099
APACHE II score ≥ 30	*n* = 39	*n* = 31	*n* = 8	
Death	29 (74.4%)	24 (77.4%)	5 (62.5%)	0.399
ΔPaO2/FiO2, median (IQR) (mmHg)	93 (23–153)	96 (23–164)	66 (26–131)	0.465
ΔLymphocyte count, median (IQR) (× 10^9^/L)	−0.06 (−0.39 to 0.16)	−0.04 (−0.27 to 0.18)	−0.28 (−0.5 to −0.03)	0.210

Abbreviations: APACHE II, Acute Physiology and Chronic Health Evaluation II; CTSSS, combination therapy of steroids and sivelestat sodium; FiO2, fraction of inspired oxygen; IQR, interquartile range; NT-proBNP, N-terminal prohormone of brain natriuretic peptide; PaO2, partial pressure of oxygen.

**Table 6:** Multivariate logistic regression analysis of risk factors for mortality in 127 patients with moderate-to-severe ARDS.

Items	B	SE	Wald	df	OR	95% CI	*p*
Duration of corticosteroid	0.328	0.134	5.982	1	1.389	1.067–1.807	0.014
Duration of sivelestat sodium	−0.336	0.116	8.415	1	0.715	0.569–0.897	0.004
ΔSOFA score	0.900	0.200	20.181	1	2.459	1.661–3.641	< 0.001
APACHE II score	0.204	0.060	11.628	1	1.226	1.091–1.379	0.001
Septic shock	2.101	0.736	8.158	1	8.176	1.933–34.573	0.004
Constant	−6.293	1.826	11.878	1	0.002	—	0.001

Abbreviations: APACHE II, Acute Physiology and Chronic Health Evaluation II; ARDS, acute respiratory distress syndrome; CI, confidence interval; OR, odds ratio; SE, standard error; SOFA, sequential organ failure assessment.

## Data Availability

The data used and analysed in this study are available from the corresponding author on reasonable request.

## Supporting Information

Supporting table caption: Table S1. Differences in changes of laboratory findings and SOFA score between the CTSSS group and the control group in each subgroup. Table S2. Univariate analysis of risk factors for in-hospital mortality in 127 patients with moderate-to-severe ARDS.
