# Supplementary material for: The Protective Effects of Sivelestat Sodium on the Basis of Corticosteroid Therapy in Patients With Moderate-to-Severe Acute Respiratory Distress Syndrome
Source: Emerg Med Int. 2025 Feb 12;2025:1824299. doi: 10.1155/emmi/1824299 (PMC11839260; doi:10.1155/emmi/1824299)
Supplement: Supporting Information — Additional supporting information can be found online in the Supporting Information section. [file 1824299.f1.docx]

| **Table S1.** Differences in changes of laboratory findings and SOFA between the CTSSS group and the control group in each subgroup. | | | | |
| --- | --- | --- | --- | --- |
| Subgroups, median (IQR) | Total | CTSSS group | Control group | *p* |
| **Age < 80 yr** | **n=103** | **n=72** | **n=31** |  |
| ΔPaO2/FiO2, mmHg | 80 (46-164) | 107 (55-176) | 64 (19-113) | 0.016 |
| ΔWhite blood cell count, ×10^9^/L | -1.22 (-4.58 to 3.76) | -0.41 (-4.35 to 3.57) | -1.96 (-5.48 to 3.96) | 0.371 |
| ΔNeutrophil count, (M±SD), ×10^9^/L | -0.40±6.86 | -0.17±6.40 | -0.93±7.99 | 0.612 |
| ΔLymphocyte count, ×10^9^/L | 0.06 (-0.17 to 0.28) | 0.12 (-0.09 to 0.37) | -0.06 (-0.41 to 0.14) | 0.016 |
| ΔHemoglobin, (M±SD), g/L | -11±20 | -10±20 | -12±19 | 0.650 |
| ΔPlatelet count, ×10^9^/L | -9 (-73 to 25) | -12 (-75 to 23) | -8 (-67 to 26) | 0.951 |
| ΔTotal bilirubin, umol/L | 0 (-4.60 to 5.60) | 0.10 (-4.52 to 7.19) | 0.89 (-4.70 to 3.61) | 0.419 |
| ΔDirect bilirubin, umol/L | 0.11 (-1.81 to 3.01) | 0.37 (-1.63 to 3.78) | -0.38 (-2.60 to 1.58) | 0.235 |
| ΔAlanine aminotransferase, U/L | 2 (-20 to 19) | 2 (-19 to 25) | 1 (-22 to 12) | 0.643 |
| ΔAspartate aminotransferase, U/L | -3 (-19 to 25) | -2 (-19 to 36) | -3 (-27 to 14) | 0.708 |
| ΔCreatinine, μmol/L | 6 (-13 to 54) | 6 (-16 to 47) | 6 (-11 to 64) | 0.725 |
| ΔAlbumin, g/L | 3.05 (-2.34 to 7.4) | 3.84 (-1.70 to 7.44) | 1.16 (-2.95 to 6.95) | 0.247 |
| ΔLactate, mmol/L | -0.68 (-1.73 to 0.31) | -0.69 (-1.70 to 0.35) | -0.67 (-1.91 to 0.27) | 0.504 |
| ΔNT-proBNP, ng/L | 98 (-1177 to 1634) | 52 (-1568 to 1518) | 127 (-582 to 1991) | 0.339 |
| ΔC-reactive protein, mg/L | -46 (-98 to -15) | -41 (-97 to -11) | -58 (-112 to -28) | 0.429 |
| ΔProcalcitonin, ug/L | -0.73 (-6.11 to 0.13) | -0.48 (-6.40 to 0.23) | -1.30 (-5.95 to 0.09) | 0.651 |
| ΔSOFA score | -1 (-3 to 2) | -1 (-3 to 1) | -1 (-3 to 2) | 0.994 |
| **Age ≥ 80 yr** | **n=24** | **n=14** | **n=10** |  |
| ΔPaO2/FiO2, mmHg | 61 (-5 to 142) | 60 (-26 to 137) | 86 (6-200) | 0.429 |
| ΔWhite blood cell count, ×10^9^/L | -2.26 (-6.69 to 1.13) | -1.09 (-9.39 to 1.86) | -2.77 (-5.55 to 1.00) | 0.907 |
| ΔNeutrophil count, (M±SD), ×10^9^/L | -2.38±5.50 | -2.83±6.42 | -1.76±4.12 | 0.648 |
| ΔLymphocyte count, ×10^9^/L | -0.05 (-0.26 to 0.13) | -0.07 (-0.39 to -0.01) | 0.12 (-0.18 to 0.29) | 0.089 |
| ΔHemoglobin, (M±SD), g/L | -23±17 | -24±18 | -20±14 | 0.559 |
| ΔPlatelet count, ×10^9^/L | -41 (-74 to -11) | -47 (-67 to -15) | -35 (-93 to 59) | 0.464 |
| ΔTotal bilirubin, umol/L | -0.93 (-5.48 to 5.43) | -1.75 (-4.54 to 2.04) | 4.86 (-7.47 to 11.85) | 0.520 |
| ΔDirect bilirubin, umol/L | 0.41 (-3.67 to 4.08) | 0.57 (-3.42 to 2.50) | 0.22 (-5.41 to 6.07) | 0.907 |
| ΔAlanine aminotransferase, U/L | -4 (-23 to 11) | -15 (-37 to 10) | -2 (-12 to 18) | 0.364 |
| ΔAspartate aminotransferase, U/L | 0 (-35 to 34) | -24 (-45 to 63) | 10 (-30 to 18) | 0.639 |
| ΔCreatinine, μmol/L | 30 (-10 to 114) | 54 (-9 to 110) | 19 (-10 to 176) | 0.861 |
| ΔAlbumin, g/L | -0.91 (-3.47 to 3.56) | -2.12 (-6.00 to 1.50) | 2.73 (-2.02 to 5.70) | 0.089 |
| ΔLactate, mmol/L | -0.68 (-1.60 to 0.30) | -0.50 (-2.41 to 0.34) | -0.83 (-1.11 to 0.27) | 0.953 |
| ΔNT-proBNP, ng/L | 0 (-1477 to 1467) | -116 (-1468 to 1194) | 402 (-3705 to 6461) | 0.598 |
| ΔC-reactive protein, mg/L | -33 (-113 to 2) | -23 (-62 to 2) | -67 (-162 to 32) | 0.482 |
| ΔProcalcitonin, ug/L | 0.97 (-0.28 to 2.58) | 0.97 (-0.63 to 3.10) | 0.85 (-0.27 to 3.50) | 0.884 |
| ΔSOFA score | 1 (-2 to 3) | 2 (0-3) | -2 (-2 to 3) | 0.239 |
| **APACHE II score <30** | **n=88** | **n=55** | **n=33** |  |
| ΔPaO2/FiO2, mmHg | 79 (30-163) | 107 (59-170) | 60 (18-134) | 0.089 |
| ΔWhite blood cell count, ×10^9^/L | -1.76 (-4.82 to 3.91) | -1.64 (-4.50 to 4.59) | -1.96 (-5.42 to 2.99) | 0.499 |
| ΔNeutrophil count, (M±SD), ×10^9^/L | -0.57±6.45 | -0.47±6.46 | -0.74±6.51 | 0.851 |
| ΔLymphocyte count, ×10^9^/L | 0.10 (-0.16 to 0.27) | 0.12 (-0.05 to 0.32) | -0.03 (-0.21 to 0.21) | 0.099 |
| ΔHemoglobin, (M±SD), g/L | -11±17 | -11±18 | -12±15 | 0.837 |
| ΔPlatelet count, ×10^9^/L | -9 (-75 to 29) | -9 (-75 to 30) | -29 (-76 to 35) | 0.803 |
| ΔTotal bilirubin, umol/L | -1.54 (-5.22 to 4.23) | -1.57 (-5.00 to 4.00) | -1.18 (-6.41 to 5.39) | 0.990 |
| ΔDirect bilirubin, umol/L | -0.27 (-2.58 to 2.15) | -0.02 (-2.00 to 2.50) | -0.50 (-4.00 to 1.55) | 0.328 |
| ΔAlanine aminotransferase, U/L | 1 (-18 to 12) | 2 (-16 to 16) | -4 (-23 to 7) | 0.311 |
| ΔAspartate aminotransferase, U/L | -3 (-27 to 15) | -5 (-24 to 17) | -3 (-29 to 15) | 0.867 |
| ΔCreatinine, μmol/L | 9 (-10 to 63) | 5 (-10 to 48) | 18 (-9 to 71) | 0.466 |
| ΔAlbumin, g/L | 3.08 (-1.96 to 6.51) | 3.90 (-1.40 to 7.36) | 1.22 (-2.69 to 6.41) | 0.250 |
| ΔLactate, mmol/L | -0.67 (-1.59 to 0.24) | -0.67 (-1.62 to 0.24) | -0.67 (-1.60 to 0.31) | 0.897 |
| ΔNT-proBNP, ng/L | 89 (-1165 to 1490) | -146 (-1524 to 1044) | 573 (-212 to 3686) | 0.006 |
| ΔC-reactive protein, mg/L | -51 (-118 to -13) | -41 (-118 to -12) | -54 (-121 to -19) | 0.853 |
| ΔProcalcitonin, ug/L | -0.30 (-4.73 to 0.23) | -0.16 (-4.87 to 0.13) | -0.33 (-4.36 to 0.98) | 0.966 |
| ΔSOFA score | -1 (-3 to 1) | -1 (-3 to 1) | -1 (-3 to 2) | 0.941 |
| **APACHE II score ≥30** | **n=39** | **n=31** | **n=8** |  |
| ΔPaO2/FiO2, mmHg | 93 (23-153) | 96 (23-164) | 66 (26-131) | 0.465 |
| ΔWhite blood cell count, ×10^9^/L | -0.88 (-5.93 to 2.52) | 0.28 (-5.93 to 2.52) | -3.76 (-6.44 to 4.85) | 0.498 |
| ΔNeutrophil count, (M±SD), ×10^9^/L | -1.24±7.14 | -0.85±6.40 | -2.75±9.91 | 0.510 |
| ΔLymphocyte count, ×10^9^/L | -0.06 (-0.39 to 0.16) | -0.04 (-0.27 to 0.18) | -0.28 (-0.50 to -0.03) | 0.210 |
| ΔHemoglobin, (M±SD), g/L | -16±26 | -15±25 | -23±27 | 0.425 |
| ΔPlatelet count, ×10^9^/L | -39 (-69 to 1) | -44 (-69 to 0) | -26 (-79 to 45) | 0.509 |
| ΔTotal bilirubin, umol/L | 3.61 (-1.50 to 14.50) | 3.60 (-2.10 to 18.20) | 3.81 (0.58-5.38) | 0.931 |
| ΔDirect bilirubin, umol/L | 1.49 (-0.42 to 8.64) | 1.49 (-1.36 to 10.40) | 2.00 (-0.10 to 7.98) | >0.999 |
| ΔAlanine aminotransferase, U/L | 1 (-21 to 45) | -2 (-26 to 45) | 8 (-1 to 87) | 0.164 |
| ΔAspartate aminotransferase, U/L | -1 (-33 to 106) | -3 (-44 to 112) | 4 (-15 to 21) | 0.808 |
| ΔCreatinine, μmol/L | 10 (-21 to 94) | 12 (-21 to 94) | -2 (-23 to 94) | 0.602 |
| ΔAlbumin, g/L | -0.24 (-4.87 to 7.40) | -0.24 (-4.87 to 7.40) | 0.55 (-5.48 to 7.57) | 0.917 |
| ΔLactate, mmol/L | -0.71 (-2.68 to 0.53) | -0.68 (-2.68 to 0.60) | -1.61 (-3.39 to 0.13) | 0.424 |
| ΔNT-proBNP, ng/L | 4 (-1664 to 5816) | 450 (-1482 to 6098) | -846 (-6917 to -75) | 0.065 |
| ΔC-reactive protein, mg/L | -41 (-70 to -5) | -36 (-60 to 17) | -64 (-85 to -35) | 0.088 |
| ΔProcalcitonin, ug/L | -0.18 (-6.50 to 5.85) | -0.18 (-7.66 to 5.85) | -0.29 (-5.27 to 4.41) | 0.889 |
| ΔSOFA score | 1 (-3 to 4) | 1 (-3 to 4) | 1 (-3 to 4) | 0.834 |
| Abbreviations: SOFA, Sequential Organ Failure Assessment; CTSSS, combination therapy of steroids and sivelestat sodium; IQR, interquartile range; PaO2, partial pressure of oxygen; FiO2, fraction of inspired oxygen; M, mean; SD, standard deviation; NT-proBNP, N-terminal prohormone of brain natriuretic peptide. | | | | |

| **Table S2.** Univariate analyses of risk factors for in-hospital mortality in 127 patients with moderate-to-severe ARDS. | | | | |
| --- | --- | --- | --- | --- |
| Parameters | Total (n=127) | Survival (n=65) | Death (n=62) | *p* |
| Age, median (IQR), y | 71 (59-78) | 68 (54-75) | 73 (65-80) | 0.005 |
| Sex |  |  |  | 0.475 |
| Male | 99 (78.0%) | 49 (75.4%) | 50 (80.6%) |  |
| Female | 28 (22.0%) | 16 (24.6%) | 12 (19.4%) |  |
| Causes of ARDS |  |  |  | 0.016 |
| Pneumonia | 85 (66.9%) | 42 (64.6%) | 43 (69.4%) |  |
| Sepsis | 27 (21.3%) | 10 (15.4%) | 17 (27.4%) |  |
| Surgical operation | 6 (4.7%) | 5 (7.7%) | 1 (1.6%) |  |
| Trauma | 5 (3.9%) | 4 (6.2%) | 1 (1.6%) |  |
| Other causes | 4 (3.1%) | 4 (6.2%) | 0 (0%) |  |
| COVID-19 | 54 (42.5%) | 24 (36.9%) | 30 (48.4%) | 0.191 |
| Severity of ARDS |  |  |  | 0.042 |
| Moderate | 67 (52.8%) | 40 (61.5%) | 27 (43.5%) |  |
| Severe | 60 (47.2%) | 25 (38.5%) | 35 (56.5%) |  |
| APACHE II score, (mean±SD) | 26.42±8.03 | 23.17±6.36 | 29.82±8.23 | <0.001 |
| SOFA score, median (IQR) | 7 (5-10) | 6 (5-9) | 8 (6-11) | 0.009 |
| Duration of corticosteroid, median (IQR), d | 5 (4-7) | 6 (4-7) | 5 (4-7) | 0.591 |
| Duration of sivelestat sodium, median (IQR), d | 4 (0-7) | 4 (0-8) | 3 (0-6) | 0.038 |
| Comorbidities |  |  |  |  |
| Hypertension | 51 (40.2%) | 30 (46.2%) | 21 (33.9%) | 0.158 |
| Cardiovascular disease | 25 (19.7%) | 9 (13.8%) | 16 (25.8%) | 0.090 |
| Diabetes | 24 (18.9%) | 14 (21.5%) | 10 (16.1%) | 0.436 |
| COPD | 15 (11.8%) | 9 (13.8%) | 6 (9.7%) | 0.467 |
| Malignancy | 12 (9.4%) | 7 (10.8%) | 5 (8.1%) | 0.602 |
| Chronic kidney disease | 10 (7.9%) | 1 (1.5%) | 9 (14.5%) | 0.008 |
| Bronchiectasia | 8 (6.3%) | 3 (4.6%) | 5 (8.1%) | 0.485 |
| Pulmonary aspergillosis | 7 (5.5%) | 3 (4.6%) | 4 (6.5%) | 0.713 |
| Complications |  |  |  |  |
| Septic shock | 59 (46.5%) | 15 (23.1%) | 44 (71.0%) | <0.001 |
| Gastrointestinal bleeding | 21 (16.5%) | 10 (15.4%) | 11 (17.7%) | 0.721 |
| Acute renal failure | 16 (12.6%) | 6 (9.2%) | 10 (16.1%) | 0.242 |
| ΔLaboratory findings, median (IQR) |  |  |  |  |
| ΔPaO2/FiO2, mmHg | 79 (27-157) | 126 (65-189) | 53 (13-115) | <0.001 |
| ΔWhite blood cell count, ×10^9^/L | -1.45 (-5.00 to 3.01) | -1.64 (-4.49 to 2.13) | -1.09 (-5.59 to 3.99) | 0.889 |
| ΔNeutrophil count, (mean±SD), ×10^9^/L | -0.77±6.65 | -1.36±6.17 | -0.16±7.11 | 0.312 |
| ΔLymphocyte count, ×10^9^/L | 0.05 (-0.19 to 0.24) | 0.14 (-0.10 to 0.47) | -0.06 (-0.28 to 0.13) | 0.001 |
| ΔHemoglobin, (mean±SD), g/L | -13±20 | -10.28±19.62 | -15.52±19.99 | 0.139 |
| ΔPlatelet count, ×10^9^/L | -26 (-73 to 17) | 1 (-45 to 55) | -45 (-79 to 0) | <0.001 |
| ΔTotal bilirubin, umol/L | -0.21 (-4.66 to 5.6) | -1.5 (-5.14 to 5.01) | 0.48 (-3.31 to 9.13) | 0.101 |
| ΔDirect bilirubin, umol/L | 0.30 (-2.02 to 3.01) | -0.15 (-3.00 to 1.68) | 0.93 (-1.35 to 8.09) | 0.019 |
| ΔAlanine aminotransferase, U/L | 1 (-20 to 12) | 2 (-21 to 12) | 1 (-19 to 34) | 0.636 |
| ΔAspartate aminotransferase, U/L | -2 (-27 to 25) | -6 (-30 to 11) | 8 (-25 to 108) | 0.008 |
| ΔCreatinine, μmol/L | 9 (-11 to 71) | -3 (-20 to 21) | 28 (-4 to 175) | 0.001 |
| ΔAlbumin, g/L | 2.48 (-2.60 to 6.51) | 3.78 (-1.97 to 6.94) | 1.38 (-3.05 to 6.16) | 0.166 |
| ΔLactate, mmol/L | -0.68 (-1.72 to 0.31) | -0.74 (-1.57 to 0.08) | 3.75 (2.29-5.11) | 0.425 |
| ΔNT-proBNP, ng/L | 80 (-1248 to 1618) | -245 (-1553 to 402) | 778 (-934 to 5480) | 0.001 |
| ΔC-reactive protein, mg/L | -42 (-98 to -12) | -59 (-137 to -22) | -36 (-82 to 5) | 0.008 |
| ΔProcalcitonin, ug/L | -0.29 (-5.07 to 1.10) | -1.77 (-7.04 to -0.01) | 0.26 (-1.48 to 5.85) | <0.001 |
| ΔSOFA score, median (IQR) | -1 (-3 to 2) | -2 (-4 to -1) | 2 (0-4) | <0.001 |
| Abbreviations: ARDS, acute respiratory distress syndrome; IQR, interquartile range; COVID-19, current coronavirus disease 2019; APACHE II, Acute Physiology and Chronic Health Evaluation II; SD, standard deviation; SOFA, Sequential Organ Failure Assessment; COPD, chronic obstructive pulmonary disease; PaO2, partial pressure of oxygen; FiO2, fraction of inspired oxygen; NT-proBNP, N-terminal prohormone of brain natriuretic peptide. | | | | |
